# The (International) American Society of Tropical Medicine and Hygiene

**DOI:** 10.4269/ajtmh.95-5ed

**Published:** 2016-11-02

**Authors:** Philip J. Rosenthal, David R. Hill, Daniel G. Bausch, Karen A. Goraleski, Stephen Higgs, Patricia F. Walker, Christopher V. Plowe

**Affiliations:** 1 Department of Medicine, University of California, San Francisco, California; 2 Frank H. Netter, MD School of Medicine, Quinnipiac University, Hamden, Connecticut; 3 Epidemic Clinical Management Unit, Pandemic and Epidemic Diseases, World Health Organization, Geneva, Switzerland and Department of Tropical Medicine Tulane School of Public Health and Tropical Medicine, New Orleans, Louisiana; 4 American Society of Tropical Medicine and Hygiene, Oakbrook Terrace, Illinois; 5 Biosecurity Research Institute, Kansas State University, Manhattan, Kansas; 6 Department of Medicine, University of Minnesota and HealthPartners Travel and Tropical Medicine Center, St Paul, Minnesota; 7 Institute for Global Health, University of Maryland School of Medicine, Baltimore, Maryland

The American Society of Tropical Medicine and Hygiene (ASTMH) was established in 1951 with the merger of the American Society of Tropical Medicine, formed by 28 American physicians in Philadelphia in 1903, and The National Malaria Committee (later Society), formed by 29 charter members in 1916. The focus of the originating societies was necessarily international from the onset. Indeed, a driving force for establishment of the American Society of Tropical Medicine was the acquisition by the United States of possessions in the Caribbean and Pacific near the turn of the twentieth century, with consequent challenges of tropical diseases such as yellow fever and malaria. Now, more than 100 years after the formation of the parent societies, and 65 years after the formation of the ASTMH, it is useful to reflect on the international scope of our Society. More than ever, our Society extends beyond the borders of the United States and welcomes contributions from those around the world interested in tropical medicine and global health.

## ASTMH Membership

**Figure 1.**
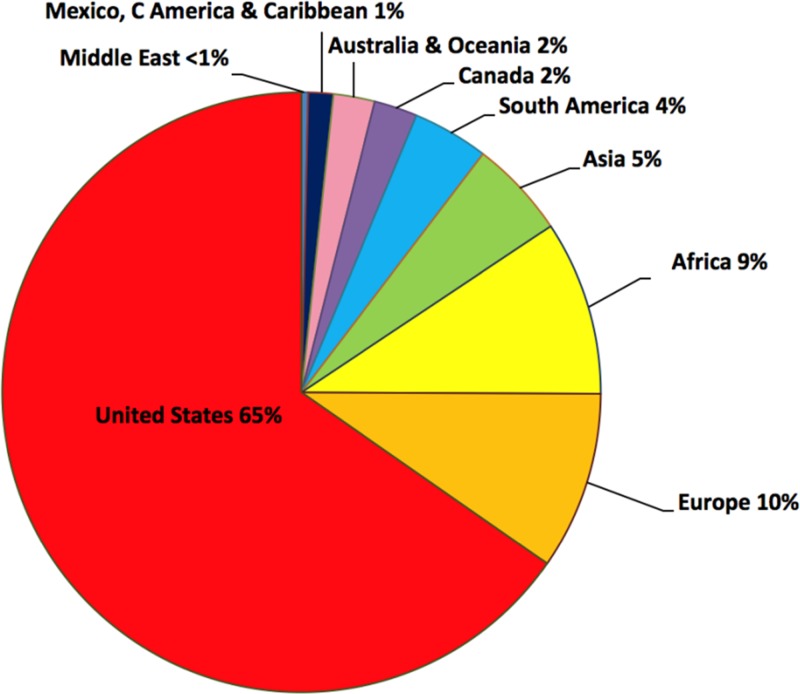
Membership of ASTMH by world region as of April 2016. Percentages are shown for 4,128 members.

The membership of ASTMH has grown steadily over the decades, reaching about 2,700 members in the first decade of the twenty-first century. Recently, the Society leadership took specific steps to increase participation of two key constituencies: trainees (undergraduate and post-doctoral students, medical residents, and fellows) and international members, particularly those from low and lower-middle income countries (LMICs). Although these constituencies were well-represented at the Annual Meeting, many attendees were not ASTMH members. For example, in 2013, 33% of Annual Meeting attendees, but only 21% of members, were from Africa, Asia, Latin America, and Europe. Thus, in 2011, the ASTMH Council voted to decrease membership dues from $65 to $15 for undergraduates and from $95 to $25 for post-doctoral students, residents, and fellows. The Council then focused on facilitating membership for international colleagues, voting in 2013 to decrease dues for members from LMICs from $230 to $25. As a likely consequence, total membership for 2016 rose to over 4,000, with trainees comprising 25% (compared with 13% prior to the dues change), and international members 35% (compared with 27% prior to the change, Figure 1

). Members from LMICs currently make up 44% of the international membership. The ASTMH also names up to five Honorary International Fellows of ASTMH (FASTMH) each year—non-Americans who have made eminent contributions to tropical medicine. To date 67 of these prestigious designations have been awarded to citizens of 30 countries.

## ASTMH Annual Meeting

**Figure 2.**
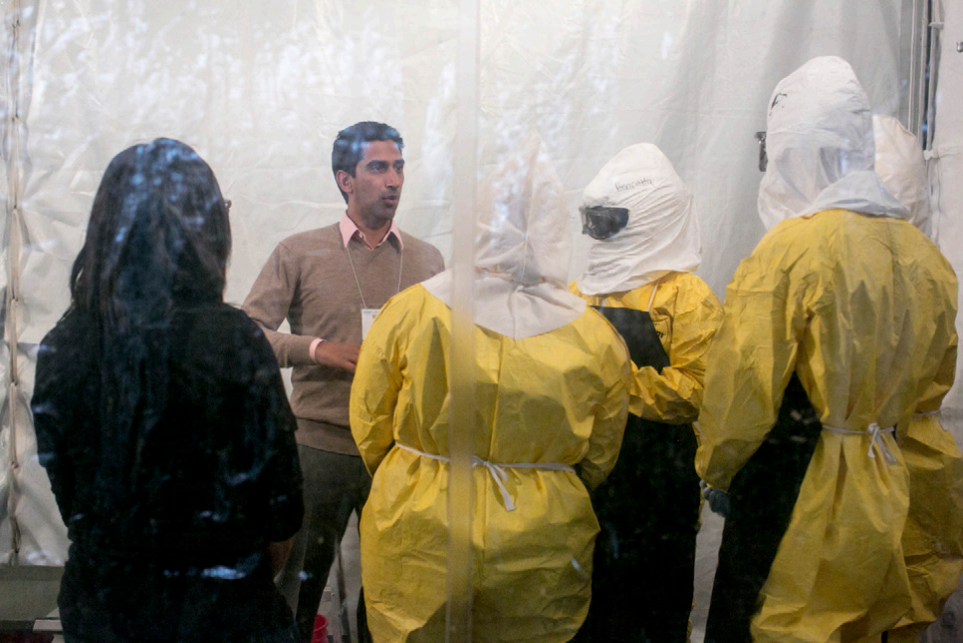
Attendees of the ASTMH annual meeting in Philadelphia, 2015, pass through a mock Ebola Treatment Unit erected to provide a real-world look into challenges of caring for patients with Ebola virus disease. The latest scientific findings on the disease were presented at numerous sessions throughout the meeting.

The flagship activity of the ASTMH is its Annual Meeting, which draws tropical medicine and global health experts from around the world representing academia, governments, non-governmental organizations, the military, industry, philanthropy, and private practice. In recent years the 5-day annual meeting has included over 600 oral presentations and 1800 posters communicating the latest breakthroughs in tropical medicine and hygiene. Presentations run the gamut from basic science to clinical practice and global health. Yearly attendance now regularly surpasses 4,000 people. In 2015, approximately one-third of meeting attendees were trainees or young investigators. In addition, approximately one third came from outside the United States, representing over 100 countries. The ASTMH places great emphasis on educational and career development opportunities for trainees, with continuing medical and veterinary education credits, pre-meeting courses with the latest updates in selected fields, “meet-the-professor” sessions, and interactive exhibits (Figure 2

) offered at the Annual Meeting. Lastly, the Society facilitates participation of young scientists and clinicians from around the world through competitive travel awards. In 2015, ASTMH made 40 such awards, 24 of which went to participants in LMICs.

## The American Journal of Tropical Medicine and Hygiene (AJTMH)

**Figure 3.**
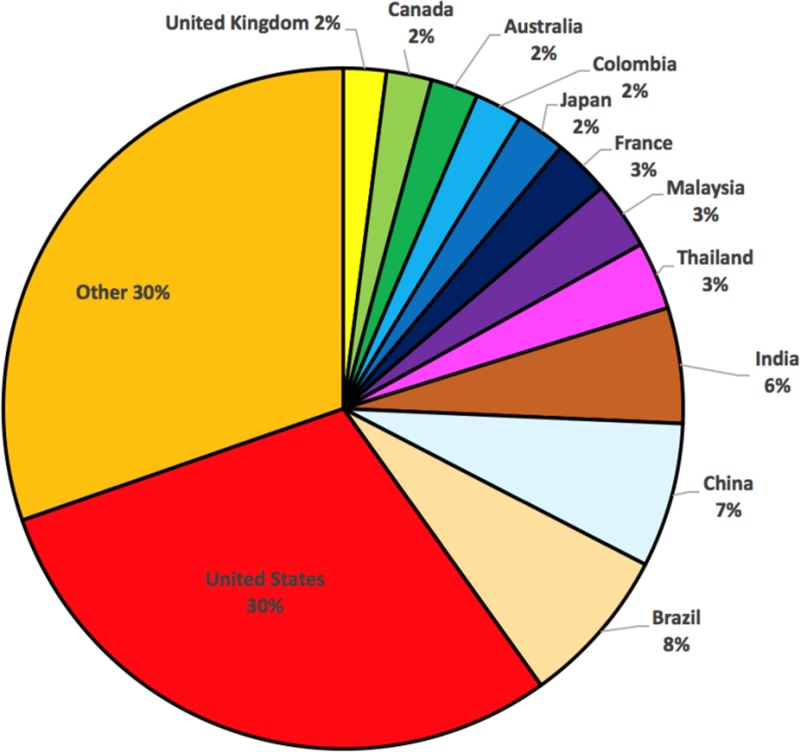
Countries of corresponding authors for manuscripts submitted to the AJTMH in 2015 (933 manuscripts).

The American Society of Tropical Medicine founded *The American Journal of Tropical Medicine* in 1921. The first issue included reports on malaria and amebiasis and a case report of yaws acquired by an American soldier in France. In early years, reports were generally from Americans conducting laboratory or animal experiments on pathogens responsible for tropical diseases or field studies in tropical regions, but there were also occasional reports from scientists working outside the United States. The AJTMH was formed in 1952 as a merger of *The American Journal of Tropical Medicine* and the *Journal of the National Malaria Society*, roughly coincident with the elimination of malaria in the United States. The first issue included papers on tropical medicine in the armed forces, dengue fever, yellow fever, scrub typhus, malaria, amebiasis, and dysentery, all authored by Americans. Over the years, international reports have increased. In 2015 only 30% of submitted manuscripts were from the United States, with manuscripts submitted from a total of 86 countries (Figure 3

). Waivers of publication fees for contributions from low income countries are available on a limited basis. The AJTMH encourages submissions from all countries of the world.

## ASTMH Around the World

In recent years, ASTMH has sought to expand opportunities for engagement internationally by partnering with members and collaborators to co-sponsor meetings and events around the world. The ASTMH President, Executive Director, Scientific Program Chair, and Council Members often attend and give presentations. ASTMH encourages international members to engage with the Society and create opportunities for participation on a local level. Examples of some recent activities are below.

### ASTMH in Peru.

For the last 6 years, ASTMH has sponsored an ASTMH Peru meeting held in Lima. The meeting was coordinated with a consortium of five Peruvian organizations: Peruvian National Institutes of Health, Peruvian Society of Infectious and Tropical Diseases, Alexander von Humboldt Tropical Medicine Institute of Universidad Peruana Cayetano Heredia, Daniel A. Carrión Tropical Medicine Institute of the Universidad Nacional Mayor de San Marcos, and the Research Unit and Training Program in Emerging Diseases and Climate Change. The 2016 meeting was dedicated to the late Alan J. Magill, past president of ASTMH and a key leader in the formation of ASTMH in Peru. The meeting was attended by over 225 people from 14 countries and offered three pre-meeting courses, including a manuscript writing workshop for trainees and young investigators, 15 oral presentations, and 67 poster presentations. The meeting was conducted in Spanish, with simultaneous English translation when needed.

### ASTMH in Kenya.

ASTMH has members from 33 countries in Africa, representing 9% of the total ASTMH membership. In 2016 ASTMH sponsored its first formal meeting in Africa, incorporated into the Sixth Kenya Medical Research Institute Annual Scientific and Health Conference, held in February 2016 in Nairobi. The conference attracted about 400 registrants from 15 countries. Oral presentations and posters were provided by young African investigators, with three awards for oral presentations and one for best poster. ASTMH members contributed to the meeting and provided formal guidance on preparation and submission of scientific manuscripts.

### ASTMH in Asia.

In 2001 the Asian Clinical Tropical Medicine Course was established in Bangkok by Mahidol University with assistance from ASTMH members. The meeting has since been held in Bangkok every 2 years. Over time, the link between Mahidol University and ASTMH has deepened. A two-week Tropical Medicine short course at Mahidol University is now offered in conjunction with a University of Minnesota online course. The course has been expanded to include a segment at Angkor Hospital for Children in Siem Reap, Cambodia. Scholarships to attend the course have been offered to physicians from Cambodia, Myanmar, and Laos.

## The ASTMH and Science Diplomacy

ASTMH recognizes that diplomacy and advocacy are often necessary for scientific advances to have their optimal impact in improving human health. An example of the Society's work in this regard was a historic meeting of diverse factions from Myanmar to cooperate on malaria elimination, held in Washington, D.C. in August 2015. More than a dozen Myanmar government health officials, military leaders, ruling and opposition politicians, and health leaders from ethnic groups in armed conflict with the government came together at a forum co-sponsored by the ASTMH, the Center for Strategic and International Studies, and the University of Maryland Institute for Global Health. The groups from Myanmar, many with a long history of antagonism and mistrust, were able to put aside their political differences in the shared interest of fighting a common enemy—malaria. The meeting culminated with public commitment from all parties to work together on a national malaria elimination campaign, including all sectors of this conflict-ridden country, irrespective of political differences, election results, or ceasefire agreements.

Another example of ASTMH science diplomacy and leadership was the Society's successful efforts with the United States State Department to enable two Iraqi scientists to present posters at the 2014 Annual Meeting. ASTMH played a similar key role in global public health détente in 2015, working with the Pan American Health Organization (PAHO) to bring three Cuban scientists to the 2015 Annual Meeting to participate in a special symposium entitled “Building Bridges Through Science and Global Health,” with participation of the National Institutes of Health Fogarty International Center, the PAHO Department of Communicable Diseases and Health Analysis, and the United States Assistant Secretary for Global Affairs. The Cuban participants were among the first to receive visas through the newly opened United States Embassy in Havana and to make formal scientific presentations in the United States in over half a century.

The ASTMH has been an international society since its inception, and it continues to strive to enhance participation by members from around the globe; increase discourse between attendees from all countries at the ASTMH Annual Meeting; build on ASTMH sponsored forums in South America, Africa, and Asia; encourage contributions to the AJTMH; and work with international partners to strengthen contributions to global scientific discourse, education, and diplomacy. We encourage our members from around the globe and contributors to ASTMH meetings and publications to spread the word. We are eager to embrace the international public health and research communities in our shared agendas to work toward a world free of tropical infectious diseases.

